# Altered medial temporal lobe subregion volumes in systemic lupus erythematosus patients with neuropsychiatric symptoms

**DOI:** 10.1186/s41927-024-00448-w

**Published:** 2025-01-26

**Authors:** Z. Makdad Najeeb, P. C. Sundgren, A. Jönsen, K. Zervides, J. Lätt, T. Salomonsson, J. Nystedt, P. Nilsson, A. Bengtsson, G. Kuchcinski, L. E. M. Wisse

**Affiliations:** 1 Department of Clinical Sciences, Diagnostic Radiology, Lund, Lund University, Lund, Sweden; 2 Department of Medical Imaging and Physiology, Skåne University hospital, Lund, Sweden; 3 Lund Bioimaging Centre, Lund University, Lund, Sweden; 4 Department of Clinical Sciences Lund, Rheumatology, Lund University, Skåne University Hospital, Lund, Sweden; 5 Department of Clinical Sciences Lund, Neurology, Lund University, Skåne University Hospital, Lund, Sweden; 6 University Lille, Inserm, CHU Lille, U1172 - LilNCog - Lille Neuroscience & Cognition, Lille, F-59000 France; 7 University Lille, CNRS, Inserm, CHU Lille, Institut Pasteur de Lille, US 41 - UAR 2014 - PLBS, Lille, F-59000 France

**Keywords:** Systemic lupus erythematosus, Neuropsychiatric lupus erythematosus, Medial temporal lobe, Hippocampus, Brodmann Area 35, Entorhinal cortex, Cognitive function, Depression, Magnetic resonance imaging

## Abstract

**Background:**

Systemic lupus erythematosus (SLE) often presents with neuropsychiatric (NP) involvement, including cognitive impairment and depression. Past magnetic resonance imaging (MRI) research in SLE patients showed smaller hippocampal volumes but did not investigate other medial temporal lobe (MTL) regions. Our study aims to compare MTL subregional volumes in SLE patients to healthy individuals (HI) and explore MTL subregional volumes in relation to neuropsychiatric SLE (NPSLE) manifestations.

**Methods:**

A total of 70 SLE patients and 25 HI underwent clinical evaluation, cognitive testing, and 3 tesla MRI imaging. T1-weighted MRI images were analyzed using the Automatic Segmentation of Hippocampal Subfields-T1 software. Analyses of Covariance were used to compare MTL subregion volumes between SLE and HI, and between NPSLE and non-NPSLE patients according to three models: the American College of Rheumatology (ACR) model defined by the ACR case definitions for NPSLE (n = 42), the more stringent Systemic Lupus International Collaborating Clinics (SLICC) B model (n = 21), and the most stringent SLICC A model (n = 15). Additionally, we explored the relation between MTL subregion volumes, cognitive functions, and depression scores in SLE patients using partial correlation analyses.

**Results:**

Significantly smaller volumes of bilateral whole hippocampus, anterior hippocampus, posterior hippocampus, and Brodmann Area 35 were demonstrated in NPSLE compared to non-NPSLE patients according to the ACR model (*p* = 0.01, *p* = 0.03, *p* = 0.04, and *p* = 0.01 respectively). The differences did not reach significance according to the SLICC B and SLICC A models. No significant differences in MTL subregional volumes between SLE patients and HI were found. Partial correlation analyses showed a significant positive correlation between left Brodmann Area 35 volume and complex attention scores in SLE patients. No significant associations between MTL subregion volumes and depression scores were demonstrated.

**Conclusions:**

NPSLE patients display significantly smaller volumes in various subregions of the MTL compared to non-NPSLE patients. These findings are suggestive of neuronal damage in MTL subregions in NPSLE patients on a group level.

**Supplementary Information:**

The online version contains supplementary material available at 10.1186/s41927-024-00448-w.

## Background

Systemic lupus erythematosus (SLE) is an autoimmune disease defined by circulating immune complexes and autoantibodies that target connective tissues, affecting a wide array of organs, and resulting in a multitude of clinical manifestations that range from mild to serious to potentially life-threatening [1]. Among the manifestations of SLE are neuropsychiatric (NP) symptoms, including headaches, cognitive impairment, epilepsy, focal neurological deficits, mood disorders and psychosis, all of which vary in presentation and intensity [2–8]. Neuropsychiatric SLE (NPSLE) can have significant repercussions on patients’ lives, resulting in both personal and societal consequences, such as reduced participation in the workforce, and an increased burden on the healthcare system [9].

Brain magnetic resonance imaging (MRI) is an important tool to enhance our comprehension of the neurobiological substrate of these symptoms. Depression and cognitive impairment stand out as two of the most common NP symptoms [6–11], prompting previous imaging studies to focus on the medial temporal lobe (MTL) and specifically the hippocampus [2, 12–19], as these regions are of crucial importance for depression and cognition, especially memory [20, 21]. Indeed, a recent meta-analysis indicated smaller hippocampal volumes and total cerebral grey matter volume in SLE patients compared to controls [12]. Additionally, two studies evaluated hippocampal volume in SLE patients longitudinally and found significant hippocampal atrophy over time [15, 17], also compared to healthy individuals (HI) [15]. However, only one of the previously mentioned studies, conducted by our group, specifically investigated NPSLE and reported significantly smaller hippocampal volumes in NPSLE patients compared to non-NPSLE patients [2].

Notably, most studies did not assess other MTL subregions than the hippocampus, even though these other regions are also known to be involved in cognitive and emotional processes [22–28]. MTL cortical regions show differential involvement in, for example, memory processes, with the anterior regions being more important for familiarity and specifically object memory, while the posterior MTL cortical regions are more involved in recollection and specifically memory of scenes [25]. Furthermore, the hippocampus is not a uniform structure; it exhibits an anterior-to-posterior differentiation through its long-axis. For example, the anterior hippocampus has been associated with emotion processing and the encoding of information, whereas the posterior hippocampus has been shown to be more involved with the retrieval of information and visual memory [19]. Different MTL subregions could therefore have a role in NPSLE.

We aim to perform a comprehensive and exploratory analysis of MTL subregional volumes in SLE. Specifically, we aim to compare MTL subregional volumes, including anterior and posterior hippocampal volumes, between SLE patients and HI, as well as between NPSLE and non-NPSLE patients. Moreover, to better understand the role of the MTL in NP symptoms in SLE, we relate MTL subregional volumes with cognitive test performance and depression scores.

## Methods

### Patients and healthy individuals

Consecutive out-care patients were asked to participate in this cross-sectional study. Patients with a confirmed diagnosis of SLE and meeting four or more Systemic Lupus International Collaborating Centers (SLICC) classification criteria for SLE [29] were eligible for inclusion. Patients were then subclassified as NPSLE and non-NPSLE according to the ACR criteria [29], which includes both CNS and PNS involvement. The inclusion criteria comprised: 1) female sex; 2) right-handedness; 3) Swedish fluency; and 4) age between 18–55 years. More information on the inclusion process is reported in Zervides et al [30].

The decision to exclusively include female participants aimed to ensure a more homogenous group. Right-handedness was specified to reduce potential impact of hemispheric differences on MRI scans. Swedish fluency was required to facilitate completion of the Swedish versions of CNS Vital Signs test (CNS-VS, a cognitive test) [31–33] and Montgomery–Åsberg Depression Rating Scale (MADRS-S, a self-reported depression scale) [34, 35]. The age range of 18–55 was chosen to encompass adult individuals and to minimize age-related MRI lesions.

Exclusion criteria for healthy individuals were male gender and age below 18 or above 55 years. Diagnosis with any autoimmune disease, such as rheumatoid arthritis, multiple sclerosis, Hashimoto’s disease etc, was ground for exclusion. Severe psychiatric conditions such as untreated depression (or high depressive scores despite medication), anxiety, burnout, bipolar disorder etc, that are significantly affecting the healthy control’s self-perceived health-related quality of life and cognitive abilities were also ground for exclusion. We further excluded patients with neurological conditions such as dementia, cancer, cerebrovascular disease, epilepsy etc, due to the possible effect on the anatomy and volume of the medial temporal lobe or the effect on the individual’s HRQOL and cognitive abilities. Inability to understand information about the study or inability to perform cognitive testing were also grounds for exclusion. Severe depressive symptoms are defined as a MADRS-S score equal to or above 35 [35], significant fatigue is defined as an FSS-score equal to or above 36 [36].

We utilized three attribution models to analyze our patient population (n = 70): the American College of Rheumatology (ACR) NP case definitions [7], and the SLICC NP attribution models [29, 37] which identify NPSLE using two models: SLICC A and SLICC B [38, 39]. The SLICC A NP attribution model includes, among other criteria, manifestations that occurred within a period of 15 months post SLE diagnosis and within the period of 6 months prior to SLE diagnosis [39]. The SLICC B NP attribution model extends to include NP symptoms still present post SLE diagnosis and within the period of 10 years prior to SLE diagnosis [39]. Minor NP symptoms, such as mild depression, anxiety, and mild cognitive dysfunction are not included in the SLICC attribution models [39]. The ACR NP case definitions and the SLICC NP attribution models are further described in Supplement I.

Ongoing glucocorticoid treatment and ongoing treatment with disease-modifying anti-rheumatic drugs (DMARDs) were registered. After giving their informed consent, all patients underwent rheumatologic and neurologic clinical examination. Assessment of disease activity was measured with the SLE Disease Activity Index-2000 (SLEDAI-2 K) [40] and accumulated organ damage was measured with the SLICC/ACR Organ Damage Index (SDI) [41].

### Neuropsychological evaluation

All subjects except one underwent the neurocognitive test battery CNS-VS to evaluate cognitive function in SLE patients under the supervision of a neuropsychologist [31–33]. The CNS-VS test battery was selected because it is short, easy to perform, and easy to supervise, with minimal error factors such as color blindness, while still covering cognitive domains relevant to SLE-associated cognitive impairment and align closely with those in the cognitive test battery recommended by the ACR, the ACR Neuropsychological Battery (ACR-NB) [42]. The CNS-VS test has been validated in traumatic brain injury, dementia and in patients with ADHD and in evaluation of cognitive dysfunction in brain tumor patients [31–33, 43–45].

The CNS-VS standardized test battery consists of seven computer-based tests (verbal and visual memory, finger tapping, symbol digit coding, the Stroop Test, a test of shifting attention and a continuous performance test) that measure ten cognitive functions (verbal memory, visual memory, psychomotor speed, reaction time, complex attention, cognitive flexibility, processing speed, executive function, simple attention, and motor speed) [31]. A more detailed description of the testing procedure that was followed is reported in Langensee et al [33]. From the cognitive functions measured, we chose to focus on following cognitive domains: visual memory (VisuM), complex attention (CompA), processing speed (ProcS), executive function (ExecF), and simple attention (SimpA), as they have been shown to be affected in SLE [46–48]. Standardized test scores generated by the CNS-VS software are calculated based on a mean of 100 and a standard deviation (SD) of 15, relative to an age-matched healthy population [31]. Cognitive impairment is defined as a standard score of ≤ 79 (corresponding with a deviation ≤–1.4 SD from the expected value), as classified by the software’s interpretation of “Moderate Deficit and Impairment Possible”. Higher scores in CNS-VS reflect better cognitive performance. Any test outcome flagged as invalid by the Validity Indicator algorithm, which identifies scores influenced by factors such as poor effort, malingering or other secondary gains, is excluded from analysis [31].

Depression score was assessed using a Swedish version of the MADRS-S [34, 35].

### MRI acquisition and analysis

Brain MRIs were obtained with a 3 tesla (T) MRI scanner (3 T Siemens MAGNETOM Skyra, Erlangen, Germany) [2, 8]. The T1-weighted magnetization-prepared rapid gradient-echo (MPRAGE) [1 mm isotropic voxel, echo time 2.54 ms/ repetition time 1900 ms/ inversion time 900 ms] was used.

**Fig. 1:**
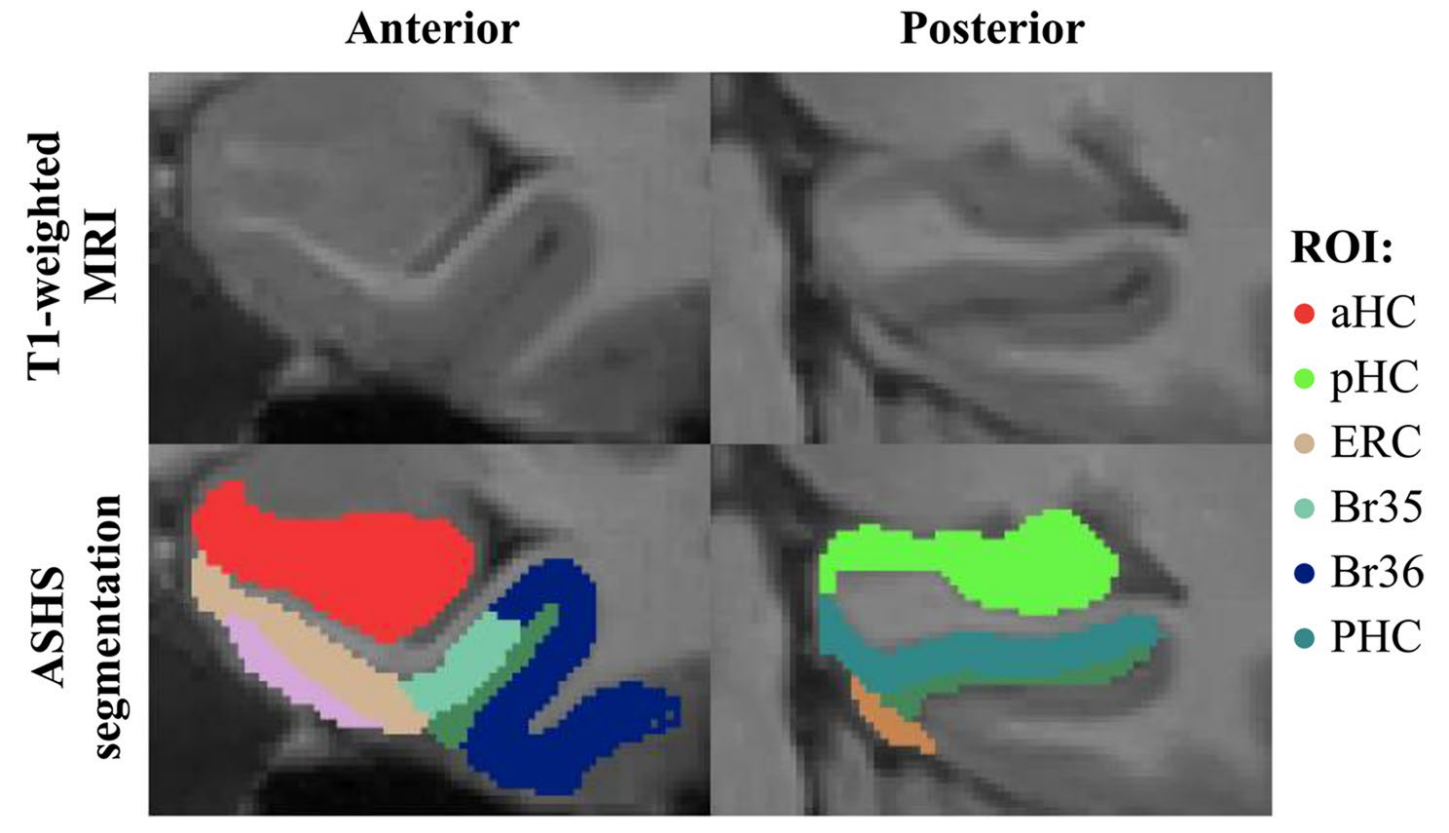
ASHS-T1 package segmentation example of medial temporal lobe subregions of an NPSLE patient. *Abbreviations* ASHS: Automated Segmentation of Hippocampal Subfields; ROI: Region of Interest; aHC: anterior Hippocampus; pHC: posterior Hippocampus; ERC: Entorhinal Cortex; BA35/36: Brodmann Area 35/36; PHC: Parahippocampal Cortex

The MPRAGE scans were segmented using Automatic Segmentation of Hippocampal Subfields-T1 (ASHS), a software program that automatically labels different MTL subregions [49, 50] (see example in Fig. 1). All segmentations were then visually checked for quality and edited in case of errors using the software application ITK-SNAP version 3.6.0 [51]. Quality checking was performed by ZMN under supervision of LEMW, blinded to the clinical status of the subject. Fifty-four cases were edited, of which 16 were HI and 38 were SLE. Volumes (in mm^3^) of the anterior hippocampus (aHC), posterior hippocampus (pHC), entorhinal cortex (ERC), Brodmann Areas 35 and 36 (BA35 and BA36), and the parahippocampal cortex (PHC) were obtained. The main analyses of each region of interest were performed on the mean volume of both hemispheres. Total intracranial volume was also extracted.

### Statistical analyses

To compare absolute MTL volumes between HI and SLE, as well as between NPSLE and non-NPSLE patients, type III Analyses of Covariance (ANCOVAs) with Tukey’s Least Significant Difference (LSD) were performed, adjusting for total intracranial volume and age as confounders.

Partial correlation analyses were conducted to evaluate the relationship between MTL subregional volumes and performance across various cognitive domains (VisuM, CompA, ProcS, ExecF, SimpA) in SLE patients, adjusting for total intracranial volume, age, and education level. Partial correlation analyses were also conducted to explore the relationship between MTL subregional volumes and depression scores (MADRS-S) in SLE patients, adjusting for total intracranial volume and age. In our partial correlation analyses, we focused only on the MTL subregions that were found to be significantly affected in NPSLE patients.

Additionally, we performed exploratory analyses for the left and right hemispheres when a group comparison or correlation between a specific subregion and cognitive domain showed a trend (*p* ≤ 0.1) towards significance.

No adjustment for disease duration was made, as age and disease duration are significantly correlated and including disease duration in the statistical models would introduce issues of multicollinearity. Given that this was an exploratory study, no correction for multiple comparisons was performed. A *p*-value < 0.05 was considered significant for all statistical analyses. All analyses were performed using IBM SPSS Statistics (Version 28.0).

## Results

### Study population

**Fig. 2:**
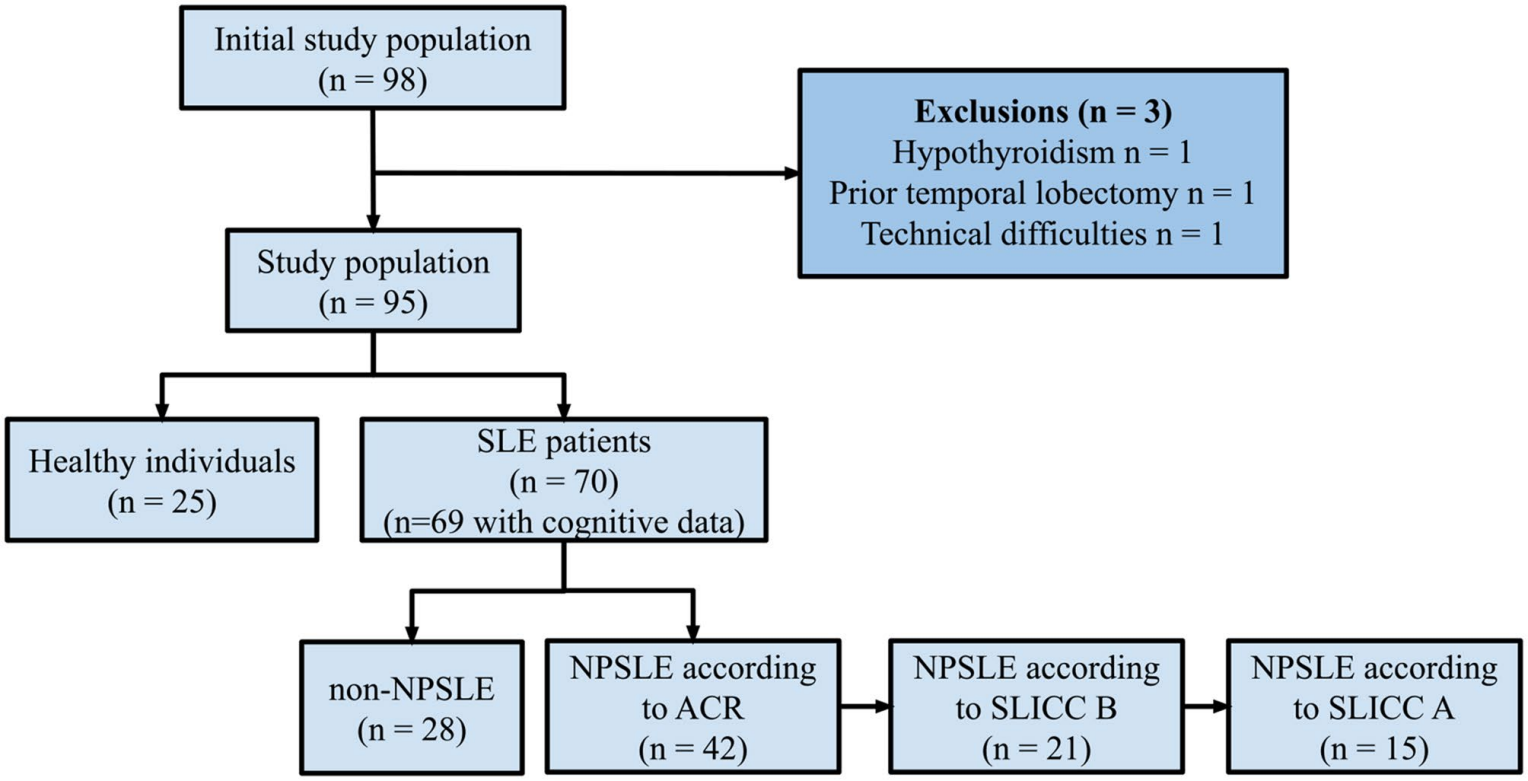
Study population categorized into different subgroups. *Abbreviations* HI: Healthy Individuals; SLE: Systemic Lupus Erythematosus; NPSLE: Neuropsychiatric SLE; SLICC: Systemic Lupus International Collaborating Clinics

A total of 98 participants were included in this study. One participant from the HI group with hypothyroidism and two participants from the patient group were excluded (one due to previous temporal lobectomy and one due to technical difficulties). Ultimately, this left us with 95 participants, 70 SLE patients and 25 HI that met the inclusion criteria for each group (see Fig. 2). The clinical characteristics of the SLE patients and HI are presented in Table 1 as well as in a previous article from our group [30]. Forty-two patients had NPSLE according to the ACR model, 21 patients according to the SLICC B model, and 15 patients according to the SLICC A model.

**Table 1:** Clinical characteristics of the study population

Subgroup		HI	SLE	Neuropsychiatric SLE according to:		
				ACR	SLICC B	SLICC A
Number (n)		25	70	42	21	15
Age [years], median (range)		37.2 (23–52)	36.0 (18–51)	36.9 (18–49)	36.9 (23–48)	39.2 (23–48)
Education level^1^	1	NA	1.4%	4.7%	4.7%	0%
	2		47.1%	61.9%	61.9%	60%
	3		50%	33.3%	33.3%	40%
MADRS-S scores (mean ± SD)^2^		2.4 ± 1.6	12.4 ± 0.9	15.8 ± 1.1	15.3 ± 1.7	16.5 ± 2.0
Disease duration [years] (mean ± SD)			11.0 ± 6.4	11.6 ± 10.6	10.6 ± 2.1	11.5 ± 5.7
SDI score (mean ± SD)^3^			0.7 ± 1.4	0.8 ± 1.4	1.1 ± 0.7	1.3 ± 0.7
SLEDAI-2 K score (mean ± SD)^4^			2.4 ± 8.5	2.6 ± 0.7	2.7 ± 0.0	3.0 ± 0.7
Anti-nuclear antibody %			98.5%	97.6%	100%	100%
Anti-double stranded DNA %			58.5%	64.2%	71.4%	66.6%
Anti-Smith antibody %			12.8%	9.5%	4.7%%	0%
Anti-phospholipid antibody %			32.8%	38.0%	42.8%	46.6%
Corticosteroid treatment %			78.6%	83.3%	80.9%	80.0%
Corticosteroid daily dose (mg/day), median (range)			5 (0–25)	5 (0–25)	5 (0–15)	5 (0–15)
DMARD treatment %	Non-antimalarial		60.0%	57.1%	47.6%	46.6%
	Antimalarial		78.6%	73.8%	80.9%	80.0%

*Abbreviations* HI: healthy individuals; ACR: American College of Rheumatology; SLICC: Systemic lupus international collaborating clinics; MADRS-S: Montgomery-Åsberg Depression Rating Scale, self-reported; SDI: SLICC/ACR damage index; SLEDAI-2 K: SLE-disease activity index 2000; DMARD: disease-modifying anti-rheumatic drug

^1^Education status is divided into 3 levels: 1 for comprehensive school (year 1–year 9) or lower, 2 for having completed upper secondary school (year 10–year 12), and 3 for having completed higher educational levels (university college/ university bachelor’s level or higher)

^2^MADRAS-S score is significantly (*p* < 0.001) higher in the SLE patients compared to HI

^3^SDI score ranges from zero (no organ damage) to 47 (highest possible level of damage)

^4^SLEDAI-2 K ranges from zero (no disease activity) to 105 (highest possible level of disease activity)

Regarding age, education level, disease duration, SDI, SLEDAI-2 K, antibody concentration Oxford, and medication, no notable differences were observed between the different groups (see Table 1). Regarding depression scores between HI and SLE subgroups, we found that MADRS-S scores were significantly (*p* < 0.001) higher in the SLE patient group (12.4 ± 0.9) compared to HI (2.4 ± 1.6).

### MTL subregional volumes in SLE compared to HI and NPSLE compared to non-NPSLE

**Fig. 3:**
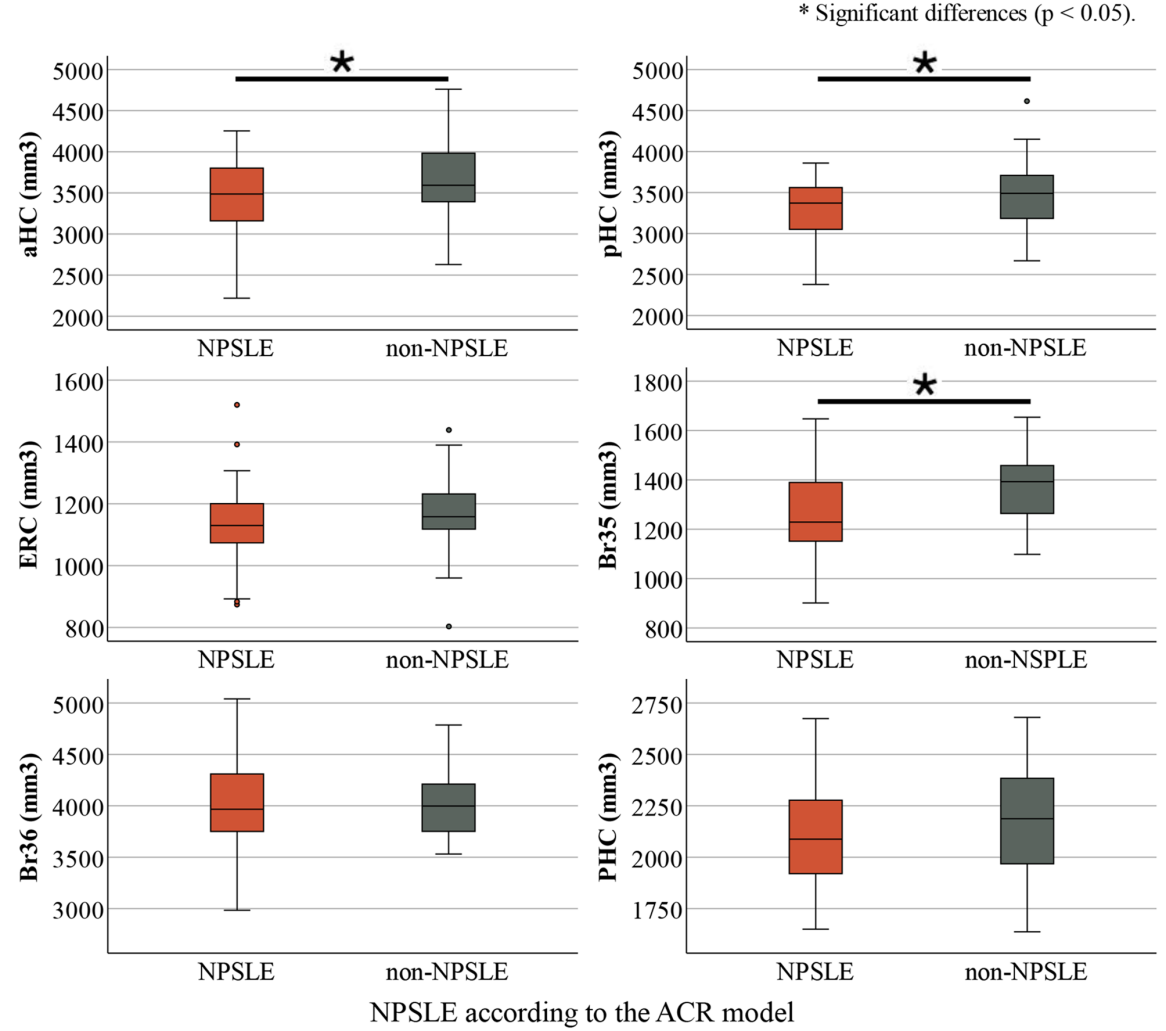
MTL subregional volumes in NPSLE and non-NPSLE patients according to the ACR model. *Abbreviations* NPSLE: neuropsychiatric SLE; HI: healthy individuals; aHC/pHC: anterior/posterior hippocampus; ERC: entorhinal cortex; BA35/36: Brodmann area 35/36; PHC: parahippocampal cortex

ANCOVAs revealed no significant differences in the volumes of subregions of the MTL between SLE patients and HI (see Table 2). Significantly smaller volumes were observed in the whole hippocampus (NPSLE: 6781.3 ± 97.3 mm^3^, non-NPSLE: 7176.3 ± 119.5 mm^3^;*p* = 0.01), the anterior hippocampus (aHC) (NPSLE: 3479.0 ± 63.9 mm^3^, non-NPSLE: 3695.0 ± 78.5 mm^3^, *p* = 0.03), posterior hippocampus (pHC) (NPSLE: 3302.2 ± 55.7 mm^3^, non-NPSLE: 3481.2 ± 68.4 mm^3^, *p* = 0.04), and BA35 (NPSLE: 1276.5 ± 20.6 mm^3^, non-NPSLE: 1361.6 ± 25.3 mm^3^, *p* = 0.01) in NPSLE compared to non-NPSLE patients according to the ACR case definition model (see Table 3 and Fig. 3).

**Table 2:** MTL subregional volumes in SLE and HI

Region of interest	Healthy individuals (mm^3^)	SLE patients (mm^3^)	HI vs SLE
	Mean ± Std. Error	Mean ± Std. Error	*p*-value
N	25	70	
HC	6918.4 ± 126.5	6946.5 ± 75.5	0.85
aHC	3499.2 ± 86.2	3570.2 ± 51.4	0.48
pHC	3419.1 ± 68.6	3376.2 ± 40.9	0.59
ERC	1112.4 ± 23.0	1150.0 ± 13.7	0.16
BA35	1274.4 ± 27.6	1311.1 ± 16.4	0.25
BA36	4057.3 ± 76.1	4016.6 ± 45.4	0.64
PHC	2116.3 ± 46.8	2133.9 ± 27.9	0.74

*Abbreviations* HC: hippocampus; aHC/pHC: anterior/posterior hippocampus; ERC: entorhinal cortex; BA35/36: Brodmann area 35/36; PHC: parahippocampal cortex

**Table 3:** MTL subregional volumes in NPSLE and non-NPSLE according to the ACR case definition model

Region of interest	ACR NPSLE (mm^3^)	Non-NPSLE (mm^3^)	NPSLE vs non-NPSLE
	Mean ± Std. Error	Mean ± Std. Error	*p*-value
N	42	28	
HC	6781.3 ± 97.3	7176.3 ± 119.5	0.01
aHC	3479.0 ± 63.9	3695.0 ± 78.5	0.03
pHC	3302.2 ± 55.7	3481.2 ± 68.4	0.04
ERC	1133.8 ± 18.6	1170.7 ± 22.9	0.22
BA35	1276.5 ± 20.6	1361.6 ± 25.3	0.01
BA36	4026.9 ± 59.9	4001.5 ± 73.6	0.79
PHC	2103.8 ± 34.9	2178.2 ± 42.8	0.18

*Abbreviations* ACR: American College of Rheumatology; NPSLE: neuropsychiatric SLE; HC: hippocampus; aHC/pHC: anterior/posterior hippocampus; ERC: entorhinal cortex; BA35/36: Brodmann area 35/36; PHC: parahippocampal cortex

A similar trend was seen between NPSLE and non-NPSLE patients according to the SLICC B and SLICC A models, however, not statistically significant (see Supplementary II and III). However, whole hippocampus and anterior hippocampus were smaller at a trend level in NPSLE compared to non-NPSLE. Upon further examination of the separate hemispheres, the left hippocampus was found to be significantly smaller in NPSLE patients (3285.3 ± 69.4 mm^3^) compared to non-NPSLE patients (3464.2 ± 45.3 mm^3^) according to the SLICC B model (*p* = 0.03), but not the right hippocampus or the left or right anterior or posterior hippocampus.

### Associations between MTL subregional volumes and cognitive performance in SLE patients

Partial correlation analyses, adjusting for age, total intracranial volume, and level of education, showed no significant association between MTL subregional volumes and cognitive performance (see Table 4), but reached a trend (*p* ≤ 0.1) for several of the analyses performed. In exploratory analyses separately for the left and right hemisphere, we found a significant positive correlation (r = 0.28, *p* = 0.02) between left BA35 volume and compA, indicating that smaller left BA35 volumes were associated with lower compA scores and thus worse complex attention. The association was not significant for right BA35 nor for any of the other regions of interest/cognitive domains that showed a trend (*p* ≤ 0.1) in Table 4.

**Table 4:** Correlating cognitive function in SLE patients to absolute volumes of aHC, pHC, HC, and BA35

Cognitive function	Region of interest	SLE patients		Cognitive function	Region of interest	SLE patients	
		Pearson’s partial correlation coefficient	*p*-value			Pearson’s partial correlation coefficient	*p*-value
Visual memory (VisuM)	aHC	−0.002	0.98	Complex attention (CompA)	aHC	0.16	0.19
	pHC	0.007	0.95		pHC	0.06	0.63
	HC	0.003	0.98		HC	0.14	0.26
	BA35	−0.009	0.94		BA35	0.20	0.10
Processing speed (ProcS)	aHC	0.19	0.11	Executive function (ExecF)	aHC	0.08	0.50
	pHC	0.09	0.46		pHC	0.004	0.97
	HC	0.18	0.15		HC	0.05	0.65
	BA35	0.21	0.09		BA35	0.14	0.24
Simple attention (SimpA^1^)	aHC	0.13	0.44	MADRS-S^2^	aHC	0.07	0.47
	pHC	0.28	0.10		pHC	−0.04	0.67
	HC	0.28	0.10		HC	0.02	0.79
	BA35	0.08	0.63		BA35	0.04	0.69

*Abbreviations* MADRS-S: Montgomery-Åsberg depression rating scale, self-reported; aHC/pHC: anterior/posterior hippocampus; BA35: Brodmann area 35

^1^SimpA has missing values in 31 SLE patients and 2 HI

^2^Partial correlations between MADRS-S and volume were not adjusted for education level

### Associations between MTL subregional volumes and depression scores in SLE patients

Partial correlation analyses showed no significant association between MTL subregional volumes and MADRS-S scores (see Table 4).

## Discussion

NPSLE patients having significantly smaller whole hippocampus, both anterior hippocampus and posterior hippocampus, and BA35 volumes compared to non-NPSLE patients aligns with our previous research [2]. Our findings suggest that the potential involvement of the hippocampus in NPSLE is not localized to the anterior or posterior portion of the hippocampus, but rather is generalized. Given the involvement of the anterior and posterior hippocampus in memory in general, but also emotional and visual memory, the observed atrophy pattern in the hippocampus coheres with the reported NP symptoms in SLE [6, 19].

BA35 was also found to be significantly smaller in NPSLE patients compared to non-NPSLE patients. BA35 is part of the perirhinal cortex, a region of the brain that plays a role in object recognition, associative and relational memory, emotional modulation of memory, expectation of reward, and integration of both concepts and percepts [52, 53]. Atrophy in this region also aligns with the observed symptom profile in NPSLE. Given the alignment of atrophy in BA35 with NPSLE symptoms, further exploration of this area could deepen our understanding of its role in NPSLE pathology.

Using a more stringent attribution model, SLICC B, NPSLE patients exhibited significantly smaller left hippocampal volumes compared to non-NPSLE patients. Using the most stringent attribution model, SLICC A, showed no significant differences between NPSLE and non-NPSLE patients. It should be noted though that the NPSLE groups defined according to these models were much smaller and it is therefore possible that the lack of significant findings was due to limited power.

Several hypotheses can be prompted regarding underlying mechanisms behind MTL atrophy in SLE. For instance, alterations in cerebral perfusion [54–58] and blood-brain barrier permeability [13, 59], reduced neurogenesis [60] and neuronal degeneration [61, 62], as well as chronic neuroinflammation [57, 63–65] or other immunological mechanisms could contribute to the observed differences.

Another hypothesis is that a more severe SLE disease with ongoing neuronal damage and increased brain pathology is present in NPLSE patients compared to non-NPSLE patients. This assumption is supported by our findings of significantly smaller volumes in NPSLE compared to non-NPSLE in various MTL subregions such as whole hippocampus, posterior hippocampus, anterior hippocampus volumes, and BA35, and the association of BA35 volume with impaired cognitive function.

No significant differences in MTL subregion volumes between SLE patients and HI were found, a result that contradicts most previous studies reporting smaller hippocampal volumes in SLE compared to HI [2, 15–17]. It is not clear why we did not observe a significant difference in hippocampal volumes between SLE patients and HI, particularly considering that a previous study involving the same participants did observe such a difference [2]. It is therefore unlikely that a difference in composition of the study population can explain this difference. One possible explanation for the difference in study findings is the use of a different approach to obtain hippocampal volumes. In the present study, a software program that automatically labels different MTL subregions (ASHS) was used to measure different subregions of the hippocampus, while in the other studies hippocampal volumes were obtained through manual delineation or other automated methods [15–17]. Note though that careful quality control and editing was performed for the current study under supervision of an expert (LEMW) with 13 years of experience with MTL segmentation in different populations, to ensure high quality volumetric data.

In a prior study, we investigated the relationship between SLE disease status and cognitive performance and found that cognitive performance is affected in both non-NPSLE and NPSLE patients [33]. However, only NPSLE patients exhibited poorer complex attention scores compared to HI, aligning with findings from multiple studies that have looked at cognitive function in SLE patients [46–48, 66, 67]. Interestingly, we found a significant correlation between the volume of the BA35 and complex attention in SLE patients. BA35, the perirhinal cortex in general and related networks have traditionally not been implicated in complex attention, future research is therefore needed to confirm this further. While an association with a memory task would have been more in line with expectations [25], the fact that both NPSLE and non-NPSLE patients were impaired in this domain may have limited the range of cognitive performance on this test and therefore power to detect such an association. It is also possible that the observed NP symptoms are more strongly related to other brain regions, such as the cerebellum or the frontal lobe, and that the MTL subregional changes are secondary and therefore less related to NP symptoms. Future research could further expand on this subject by looking into other brain subregions, such as the cerebellum where changes between SLE and healthy individuals have been demonstrated.

Our analyses, in accordance with prior research [68], revealed no significant correlations between MTL subregion volumes and depressive symptoms in SLE patients. This may be explained by the fact that the severity of depressive symptoms in our SLE cohort was generally mild. In an SLE population with a wider range of depressive symptoms, an association with MTL structural measures may still be observed. Additionally, we used a self-report measure, instead of an assessment by a psychiatrist, which may be less precise.

### Strengths and limitations

Our study population is a well characterized SLE population and the first to investigate MTL subregions in SLE patients. The use of advanced imaging techniques, such as 3 T MRI and the ASHS segmentation, enhances the precision of our volumetric measurements.

One limitation is that we only included female participants, aligning with the higher prevalence of SLE in women [69], between 18 and 55 years. This limits the generalizability of our results to SLE patients outside this age range and to male SLE patients.

While this is a large study compared to previous work, the sample size is still relatively small, especially in the subgroups (SLICC-B: n = 21 and SLICC-A: n = 15) as mentioned above. This may have resulted in limited statistical power.

Finally, we did not account for potential confounders such as treatment or disease duration in our analyses. However, we based this decision on previous research that showed no significant relationship between factors such as cumulative corticosteroid dose and cerebral and corpus callosum volumes [70].

## Conclusions

NPSLE patients display significantly smaller volumes in various subregions of the MTL compared to non-NPSLE patients. These findings are suggestive of neuronal damage in MTL subregions in NPSLE patients on a group level.

## Electronic supplementary material

Below is the link to the electronic supplementary material.

Supplementary Material 1

Supplementary Material 2

Supplementary Material 3

Supplementary Material 4

## Data Availability

All data generated and analyzed during this study are included in the article and its supplementary information files.

## Abbreviations

SLE: Systemic lupus erythematosus
NPSLE: Neuropsychiatric systemic lupus erythematosus
MRI: Magnetic resonance imaging
MTL: Medial temporal lobe
HI: Healthy individuals
SLICC: Systemic lupus erythematosus international collaborating clinics
CNS-VS: Central nervous system vital signs
MADRS-S: Montgomery-Åsberg Depression Rating Scale - Self-rated version
ACR: American College of Rheumatology
DMARD: Disease-modifying anti-rheumatic drug
SLEDAI-2K: SLE disease activity index 2000
SDI: SLICC/ACR damage index
VisuM: Visual memory
CompA: Complex attention
ProcS: Processing speed
ExecF: Executive function
SimpA: Simple attention
SD: Standard deviation
T: Tesla
MPRAGE: Magnetization-prepared rapid gradient-echo
ASHS: Automatic segmentation of hippocampal subfields
HC: Hippocampus
aHC/pHC: Anterior/Posterior hippocampus
ERC: Entorhinal cortex
BA35/ BA36: Brodmann Areas 35/ 36
PHC: Parahippocampal cortex
ANCOVA: Analysis of covariance
