# Supplementary material for: Altered medial temporal lobe subregion volumes in systemic lupus erythematosus patients with neuropsychiatric symptoms
Source: BMC Rheumatol. 2025 Jan 26;9:10. doi: 10.1186/s41927-024-00448-w (PMC11765921; doi:10.1186/s41927-024-00448-w)
Supplement: Supplementary file 4 — Supplementary Material 4 [file 41927_2024_448_MOESM4_ESM.docx]

**Supplement IV:** MTL subregional volumes between (A) SLE patients and healthy individuals, (B) NPSLE and non-NPSLE patients according to SLICC B, and (C) NPSLE and non-NPSLE patients according to SLICC.

1.
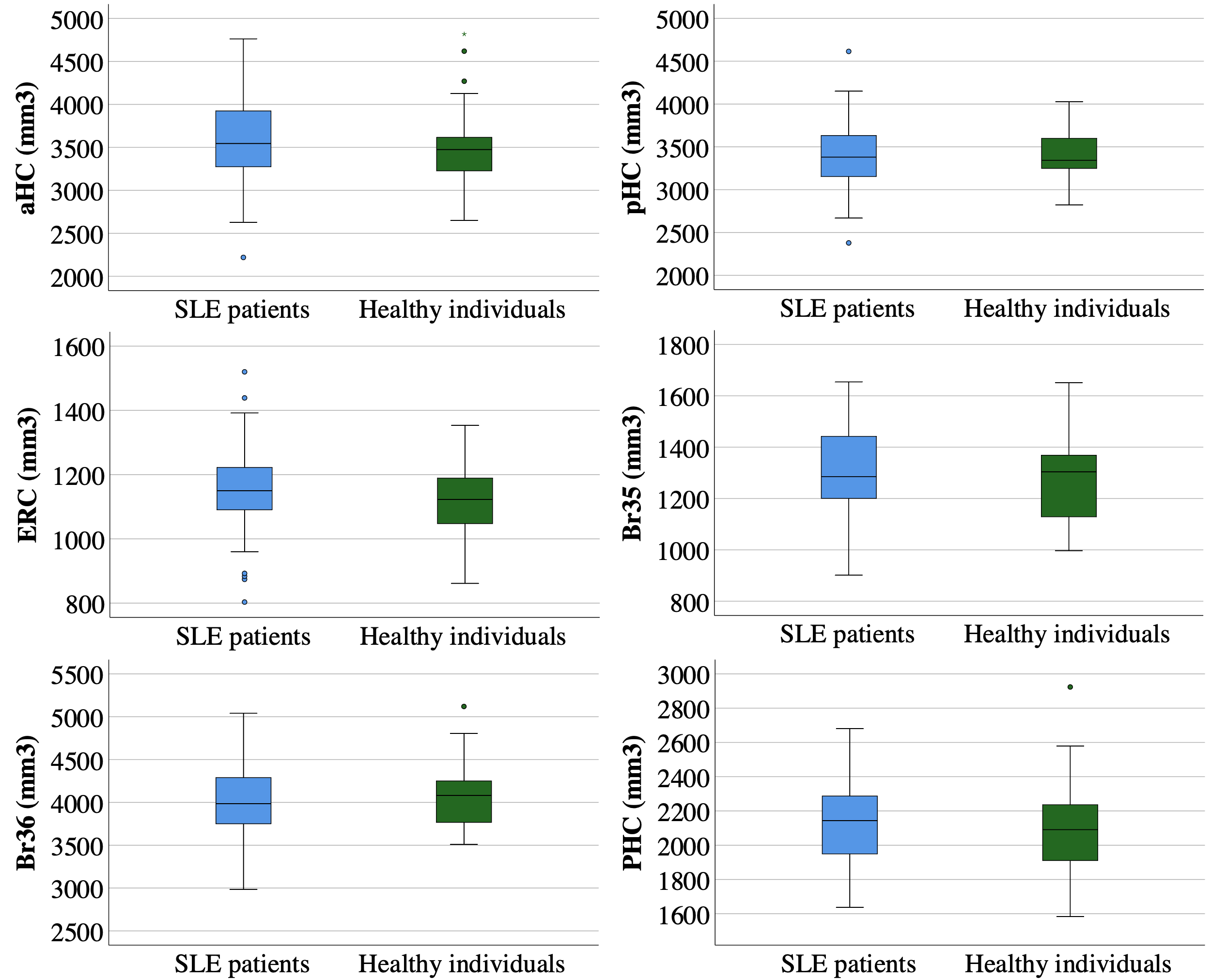

2. **
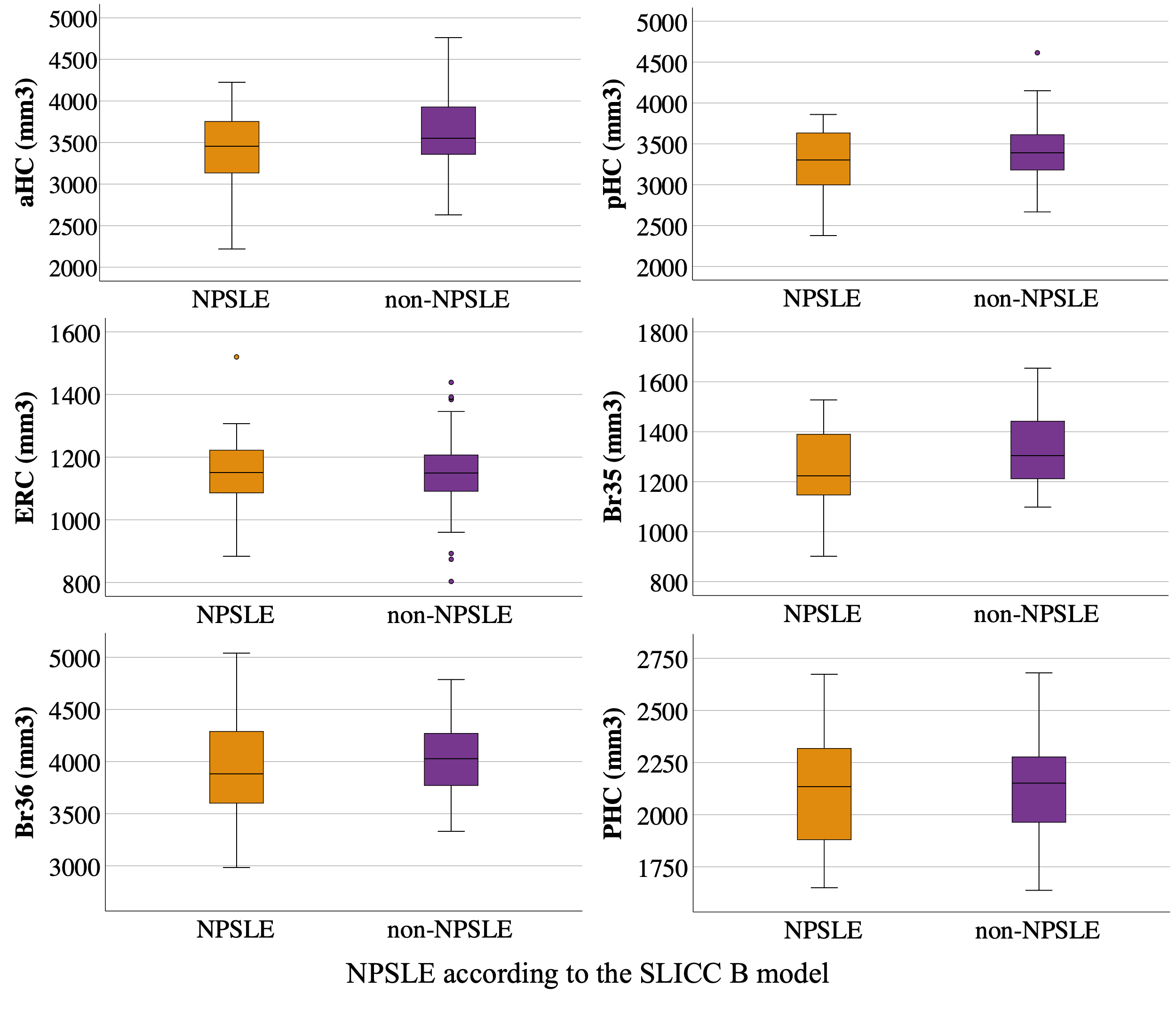
**
3. **
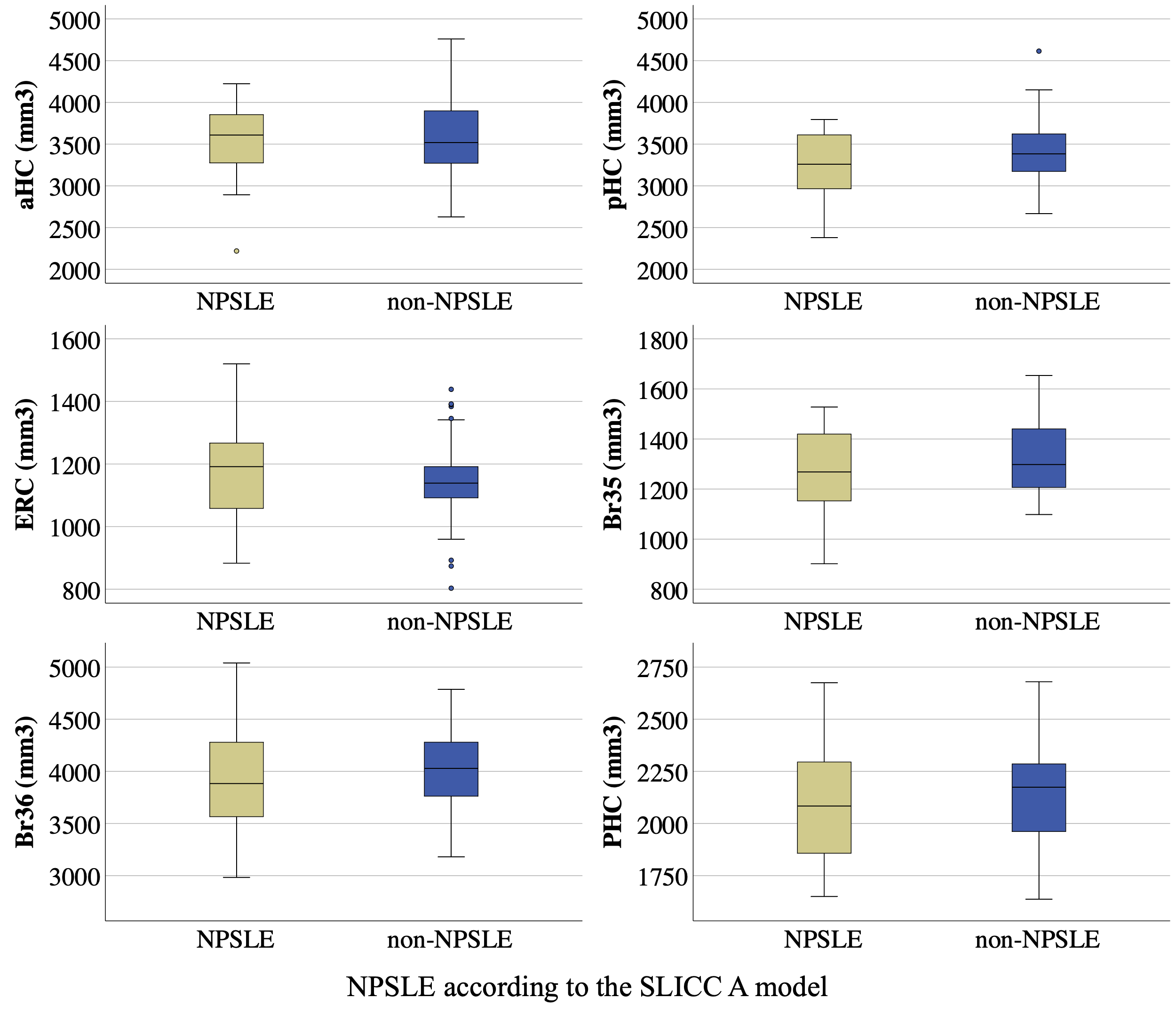
**
